# Relationships between the menstrual cycle and neuropsychiatric and physical symptoms in females with Tourette syndrome

**DOI:** 10.3389/fneur.2025.1500766

**Published:** 2025-02-11

**Authors:** Daisy T. Noriega-Makarskyy, Evan Realbuto, Ariadne Kaylor, Lisa Osiecki, Angela Essa, Dongmei Yu, Cornelia Illmann, Carol A. Mathews, Jeremiah M. Scharf

**Affiliations:** ^1^Psychiatric and Neurodevelopmental Genetics Unit, Center for Genomic Medicine, Massachusetts General Hospital, Boston, MA, United States; ^2^Stanley Center for Psychiatric Research, Broad Institute of MIT and Harvard, Cambridge, MA, United States; ^3^Department of Psychiatry, University of Florida, Gainesville, FL, United States; ^4^Genetics Institute, University of Florida, Gainesville, FL, United States; ^5^Center for OCD, Anxiety and Related Disorders, McKnight Brain Institute, University of Florida, Gainesville, FL, United States; ^6^Department of Psychiatry, Massachusetts General Hospital, Harvard Medical School, Boston, MA, United States; ^7^Department of Neurology, Brigham and Women’s Hospital, Harvard Medical School, Boston, MA, United States; ^8^Department of Neurology, Massachusetts General Hospital, Harvard Medical School, Boston, MA, United States

**Keywords:** Tourette syndrome, tic disorders, tic fluctuations, menstrual cycle, female, sex hormones, neurosteroids, allopregnanolone

## Abstract

**Background:**

The effects of the menstrual cycle on neuropsychiatric and physical symptoms have been examined in multiple psychiatric illnesses, but research on Tourette syndrome (TS) and menstruation is limited and inconclusive. One study published in 1992 reported that 34% of female respondents experienced tic fluctuations with their menstrual cycles; however, a subsequent 2001 study found no significant relationship between menstrual cycle-related hormonal changes and tic symptoms across participants. There has been no further published exploration of this topic in the intervening 20+ years, and thus these discrepant results have not been reconciled. The current study aimed to assess tic changes across the menstrual cycle and to explore clinical predictors of tic fluctuations in adult females with TS.

**Methods:**

An online survey was completed by 112 of 315 eligible female adults with TS. Respondents were asked to share their age of TS symptom onset, history of OCD and ADHD diagnoses, and current tic symptoms and severity. Participants also retrospectively reported their experiences with fluctuations in tics and other physical and psychiatric symptoms over the course of the menstrual cycle.

**Results:**

26% of the 112 respondents endorsed tic changes in relation to their menstrual cycles. Univariable and multivariable logistic regression demonstrated that higher current tic severity and impairment as well as co-occurring cycle-related mood and anxiety changes significantly predicted the presence of self-reported tic fluctuations during the menstrual cycle.

**Discussion:**

Results suggest that some females with TS experience changes in tic symptoms during their menstrual cycles, although future research is required to clarify the complex relationships between the menstrual cycle, tics, and other psychiatric symptoms. The low response rate, retrospective recall of symptoms, and lack of information about hormonal influences such as contraceptives and menopause are notable study limitations.

## 1 Introduction

Tourette syndrome (TS) is a childhood-onset neurodevelopmental disorder characterized by multiple involuntary and repetitive motor movements and vocalizations (tics) that wax and wane over the course of at least a year. Tics manifest in different forms with wide variation in severity and impairment (1), and some research suggests that TS presentation differs between males and females (2, 3). Existing literature on TS and other neurodevelopmental disorders indicates a male-to-female skew, with a ratio of approximately 4:1 for TS and 1.5:1 for chronic/persistent tics (4). TS affects 0.6–0.8% of children, with approximately 20% continuing to experience tics in adulthood (5, 6). However, males are more likely to experience tic remission in adulthood, while women with TS tend to have increased tic-related impairment as adults (3, 7, 8). Despite this, women with tic disorders remain an understudied population (8).

Although anecdotal reports by individuals with TS have described tic fluctuations with the menstrual cycle, studies examining the presence and prevalence of catamenial (menstrual cycle-related) tic fluctuations and their clinical correlates are lacking, with only two published articles to date. Schwabe and Konkol (9) reported that 16 of 47 participants (34%) who responded to a self-report questionnaire endorsed tic changes over the menstrual cycle, with increased tics reported by 12 participants (26%) during the premenstrual phase, 8 (17%) during menstruation, and 0 during the postmenstrual phase (9). However, the study did not explore clinical factors associated with these tic changes, such as tic severity at baseline or the presence of fluctuations in co-occurring neuropsychiatric disorders, and has not yet been replicated. Kompoliti et al. (10) followed eight participants with regular menstrual cycles over 5 weeks, measuring weekly serum estrogen and progesterone levels. Estrogen and progesterone fluctuations were consistent with normal menstrual cycles, and no significant group-level associations were observed between levels of either hormone and observed tic counts, self-reported Yale Global Tic Severity Scale (YGTSS) scores, or self-reported Yale–Brown Obsessive-Compulsive Scale (Y-BOCS) scores. Only one of the eight participants reported menstrual cycle-related tic fluctuations, and this patient’s estrogen levels demonstrated a significant inverse correlation with tic counts at each visit (10). However, the generalizability of this observation was limited by the small sample size.

Though research on TS and the menstrual cycle is limited, some researchers have investigated catamenial symptom changes in disorders that often co-occur with TS. One study on obsessive-compulsive disorder (OCD), a disorder that is present in approximately 57% of females with TS and is thought to have overlapping genetic susceptibility with tic disorders (11), identified OCD symptom exacerbation in the premenstrual period in 49 out of 101 (49%) study participants (12). Similarly, Labad et al. (13) reported increased premenstrual OCD symptoms in 9 of 45 (20%) participants and found a significant association between OCD symptom exacerbation and premenstrual mood symptoms (13). There has also been recent interest in the effects of the menstrual cycle on attention-deficit/ hyperactivity disorder (ADHD), another disorder that co-occurs in approximately 40% of females with TS (11). For example, Roberts et al. (14) found that when participants experienced decreased estradiol along with increased progesterone or testosterone, they tended to experience increased ADHD symptoms the following day. For those high in specific ADHD-related traits such as sensation seeking and positive urgency, increased ADHD symptoms were reported during the follicular and postovulatory phases (14).

Catamenial symptom fluctuations have also been observed across other neuropsychiatric disorders. In a literature review published by Pinkerton et al. (15), two studies reported premenstrual exacerbation of panic attacks in 18 out of 50 (36%) individuals with panic disorder (16) and in 14 out of 43 (33%) individuals with panic disorder and/or agoraphobia with a history of panic attacks (17). Another systematic review by Green and Graham et al. (18) identified two studies demonstrating that, during the premenstrual period, 15 out of 19 (79%) participants with panic disorder reported elevated anxiety symptoms, 11 out of 19 (58%) reported more frequent panic attacks (19), and 39 out of 94 (41%) reported increased panic disorder symptoms (20). In a recent review of premenstrual dysphoric disorder (PMDD), Lanza di Scalea and Perlstein (21) noted that 20–25% of female adults experience moderate to severe premenstrual symptoms, even though only 5% meet formal criteria for PMDD (21). Other studies demonstrate that although most individuals do not experience catamenial exacerbation of neuropsychiatric disease, symptoms of anxiety, bipolar disorder, eating disorders, and PMDD can all be affected by the menstrual cycle in a subset of females with these illnesses (15).

These data indicate that there may be common factors amongst those who experience catamenial changes in neuropsychiatric symptoms. In addition, the prevalence of menstrual cycle-related symptom fluctuations in other neuropsychiatric disorders suggests that these fluctuations may also occur in individuals with TS. However, as noted, only two studies have investigated catamenial symptom fluctuations in TS, both with small sample sizes and limited results. Thus, the current study aimed to (a) assess the presence and prevalence of self-reported fluctuations in tic symptoms with the menstrual cycle in adult females with Tourette syndrome, and (b) examine which clinical factors might be associated with the presence or absence of these catamenial tic fluctuations.

## 2 Materials and methods

### 2.1 Participants

315 eligible cisgender female adults who previously participated in TS genetic studies were recontacted for the current follow-up study an average of 7 years after initial enrollment (22–24). Of the 315 eligible participants, 112 (36%) provided consent and completed the self-report survey on tic fluctuations and the menstrual cycle. 203 participants were lost to follow-up. Participants who reported intellectual disability, epilepsy, or other genetic or neurological disorders that could confound a TS diagnosis were excluded from the initial genetic studies and thus also from this study (22). All study procedures were reviewed and approved by the Mass General Brigham Institutional Review Board.

### 2.2 Measures

#### 2.2.1 Menstruation questionnaire

**Figure 1:**
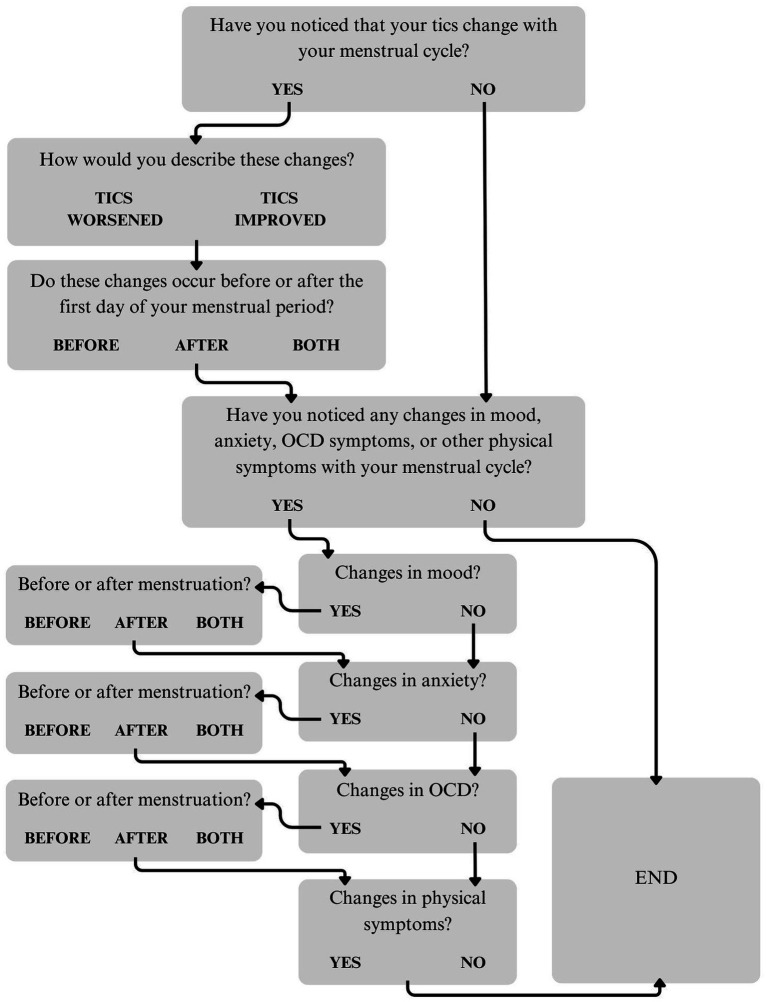
Flowchart of the menstruation questionnaire.

A questionnaire about the menstrual cycle and symptom fluctuations was created by a team of clinicians and researchers as part of a larger survey. The questionnaire first asked participants to share whether they had noticed that their tics fluctuate with their menstrual cycle. Answer options were “Yes,” “No,” or “Prefer not to answer.” Those who endorsed catamenial tic fluctuations were asked about the direction of these changes (“Tics worsened,” “Tics improved,” or “Other”). Participants who selected “Other” were asked to describe these changes in an open-ended text box. The questionnaire then asked about the timing of these changes in relation to the first day of menstruation (“Before,” “After,” or “Both before and after”). Participants were asked to specify the number of days before and/or after menstruation onset that they experienced tic fluctuations (Supplementary Figure 1). Those who selected “Both before and after” were given an open-ended text box to provide additional information. Next, participants were asked to report whether they had noticed changes in mood, anxiety, OCD, or other physical symptoms with their menstrual cycle (“Yes,” “No,” or “Prefer not to answer”). For those who endorsed mood, anxiety, and/or OCD symptom fluctuations, subsequent questions asked about the timing of these changes (Supplementary Figures 2–4). Participants who endorsed fluctuations in physical symptoms were asked to provide more information in an open-ended text box. All participants who endorsed tic fluctuations were also given an opportunity at the end of the questionnaire to provide additional information about changes in their tics, mood, anxiety, OCD symptoms, and/or other physical symptoms. See Figure 1 for a flowchart of the menstruation questionnaire.

Participants were not asked about hormonal factors that influence the menstrual cycle such as contraceptives or menopause. However, participants were told, “If you are currently menopausal, please consider any changes in your tics that you experienced around the time of your period BEFORE going through menopause.” In addition, participants were given multiple opportunities to share details about their catamenial tic and other symptom fluctuations through optional open-ended response boxes. In their open-ended responses, six participants mentioned taking birth control. To assess whether these participants’ responses significantly influenced the study’s results, univariable analyses were run following the same methods as described in section 2.3, but with these six participants removed from analysis. This sensitivity analysis did not demonstrate substantial differences from the univariable analyses using the full sample (Supplementary Table 1). In addition, it was not possible to assess whether the remainder of the cohort was taking or had a history of taking hormonal birth control. Therefore, these six participants were included in the final sample.

#### 2.2.2 Tic severity and impairment

A modified self-report version of the Yale Global Tic Severity Scale (YGTSS) was used to collect data on each subject’s current (within the past 6 months) tic symptoms, severity, and impairment (23, 25). Tic severity (YGTSS Total Tic Score) and global impairment (YGTSS Impairment Score) were each scored on a scale of 0–50. Each outcome was analyzed as a separate predictor, as they are believed to capture different aspects of TS disease symptomology (26). The YGTSS Total Tic Score is based on the number, frequency, intensity, complexity, and disruption of motor and vocal tics, while the YGTSS Impairment Score measures the inability to perform age-appropriate routine tasks in various domains of life and is thought to be strongly influenced by the presence of co-occurring neuropsychiatric conditions such as OCD and ADHD (1, 26). Participants were also asked to report how old they were when their tics first started and whether they were currently taking medication for their tics.

#### 2.2.3 OCD and ADHD diagnoses

Participants were asked to report whether they had ever been diagnosed with OCD or ADHD by a clinician.

#### 2.2.4 Demographics

Demographic variables (race, ethnicity, age) were collected in the original TS genetic studies.

### 2.3 Statistical analyses

Analyses were conducted using Stata, RStudio, and Excel. The number and proportion of adult female participants reporting menstrual tic fluctuations were calculated. Univariable associations between the presence/absence of menstrual-cycle related tic fluctuations and candidate predictor variables (OCD and ADHD diagnoses; tic medication status; catamenial symptom changes in mood, anxiety, OCD, and physical symptoms; YGTSS Total Tic Score, YGTSS Impairment Score; current age; and age of tic onset) were each assessed using logistic regression. Odds ratios and 95% confidence intervals (CIs) were calculated for each univariable association. Variables with univariable *p* < 0.10 were considered for inclusion in a multivariable logistic regression model. Correlations between related predictor variables were assessed. Predictor variables that were at least moderately correlated with each other (*τ* or *r* > 0.5) were not included in the same multivariable model. Multivariable models were fit with catamenial tic changes as the outcome and the candidate variables that met the criteria above included as predictors. The multivariable models were evaluated.

## 3 Results

### 3.1 Sample characteristics

118 of 315 (37%) eligible adult females from the initial TS genetic studies completed the follow-up survey. Six of these participants did not answer questions regarding menstrual cycle-related tic fluctuations and were excluded from the analyses, resulting in a final sample of 112. The mean age of participants was 33.9 years (SD = 13.0, range = 18–70). 96% of participants self-identified as White and 4% as more than one race. 97% self-identified as non-Hispanic, 2% as Hispanic, and 1% unknown (Table 1).

**Table 1:** Sample characteristics.

Demographic Characteristics			
Race	96% White		
Ethnicity	97% non-Hispanic		

Categorical Characteristics	Yes	No	Unsure/Prefer Not to Answer
OCD diagnosis	50 (45%)	54 (48%)	8 (7%)
ADHD diagnosis	36 (32%)	76 (68%)	0 (0%)^*^
Mood, anxiety, OCD, or physical changes	70 (63%)	42 (38%)	0 (0%)^*^
Mood changes	67 (60%)	43 (38%)	2 (2%)
Anxiety changes	48 (43%)	61 (54%)	3 (3%)
OCD changes	20 (18%)	90 (80%)	2 (2%)
Physical changes	35 (31%)	75 (67%)	2 (2%)

Continuous Characteristics	Mean	SD	Total *n*
TS age of onset (years)	7.1	3.1	106
Age at recontact (years)	33.9	13.0	112
YGTSS Total Tic Score (0–50)	19.3	10.5	112
YGTSS Impairment Score (0–50)	13.7	12.7	112

OCD, obsessive-compulsive disorder; ADHD, attention-deficit/hyperactivity disorder; TS, Tourette syndrome; YGTSS, Yale Global Tic Severity Score.

* These items had no option for “unsure” or “prefer not to answer.”

Participants’ mean age of tic onset was 7.1 years (SD = 3.1). The mean current YGTSS Total Tic Score was 19.3 (SD = 10.5), and the mean current YGTSS Impairment Score was 13.7 (SD = 12.7), both consistent with mild tic severity. 45% of participants reported that they had been diagnosed with OCD, and 32% reported that they had been diagnosed with ADHD (Table 1).

### 3.2 Frequency and characteristics of catamenial tic fluctuations

29 of the 112 participants (26%) reported experiencing menstrual cycle-related tic changes. 93% of these participants endorsed tic worsening, while the remaining 7% reported experiencing tic changes, but did not clarify whether their tics increased or decreased. Of the participants who reported tic changes, 66% reported experiencing changes exclusively before the first day of menstruation, 31% experienced changes both before and after menstruation onset, and 3% experienced changes exclusively after the initiation of menstruation (Supplementary Figure 1).

### 3.3 Menstrual-related psychiatric and physical changes

Across the entire sample, 70 of the 112 participants (63%) reported changes in mood, anxiety, OCD, or other physical symptoms in relation to their menstrual cycles. 60% reported mood changes, 43% anxiety changes, 18% OCD changes, and 31% other physical changes (Table 1). Participants were not asked about the direction of these changes (improvement vs. worsening). Participants who endorsed physical changes were given the option to provide more information in an open-ended text box. 34 participants responded, with the most commonly reported symptoms being cramps (10 participants), headaches/migraines (9 participants), fatigue/tiredness (8 participants), and bloating (8 participants). For each variable, responses of “unsure” or “prefer not to answer” were removed prior to univariable analyses.

### 3.4 Univariable analyses

The relationship between menstrual cycle-related tic fluctuations and each candidate predictor variable was assessed using simple logistic regression. Of the 70 participants who reported changes in mood, anxiety, OCD, or physical symptoms, 37% also reported catamenial tic changes. In contrast, of the 42 participants who did not report mood, anxiety, OCD, or physical changes, only 7% endorsed menstrual cycle-related tic fluctuations (OR = 7.68, *p* = 0.002). Participants who reported catamenial mood (OR = 5.44, *p* = 0.004), anxiety (OR = 4.73, *p* = 0.001), and OCD (OR = 5.25, p = 0.002) symptom changes were more likely to endorse catamenial tic changes. Those with higher YGTSS Total Tic (OR = 1.07, *p* = 0.004) and Impairment (OR = 1.05, *p* = 0.003) Scores were also significantly more likely to report menstrual cycle-related tic fluctuations. There were no significant differences in likelihood to report catamenial tic changes by current tic medication status, OCD diagnosis, ADHD diagnosis, physical changes, age of tic onset, or age at recontact (Table 2).

**Table 2:** Univariable predictors of menstrual cycle-related tic changes.

Categorical Measures		% Reporting Tic Changes	OR	95% CI	*p*-value
Currently taking medication for tics?	Yes (*n* = 30)	33%	1.66	0.65–4.11	0.28
	No (*n* = 82)	23%			
Diagnosed with OCD?	Yes (*n* = 50)	28%	1.36	0.56–3.36	0.50
	No (*n* = 54)	22%			
Diagnosed with ADHD?	Yes (*n* = 36)	22%	0.75	0.28–1.85	0.54
	No (*n* = 76)	28%			
Reported mood, anxiety, OCD, or physical changes?	Yes (*n* = 70)	37%	7.68	2.46–33.98	0.002^*^
	No (*n* = 42)	7%			
Reported mood changes?	Yes (*n* = 67)	36%	5.44	1.90–19.75	0.004^*^
	No (*n* = 43)	9%			
Reported anxiety changes?	Yes (*n* = 48)	42%	4.73	1.91–12.71	0.001^*^
	No (*n* = 61)	13%			
Reported OCD changes?	Yes (*n* = 20)	55%	5.25	1.89–15.06	0.002^*^
	No (*n* = 90)	19%			
Reported physical changes?	Yes (*n* = 35)	31%	1.56	0.63–3.81	0.33
	No (*n* = 75)	23%			

Continuous Measures	Reported Tic Changes: Mean (SD)	Did Not Report Tic Changes: Mean (SD)	OR	95% CI	*p*-value
TS age of onset (years)	7.0 (2.6)	7.1 (3.2)	0.99	0.86–1.14	0.89
Age at recontact (years)	31.8 (9.6)	34.6 (13.9)	0.98	0.95–1.02	0.32
YGTSS Total Tic Score (0–50)	24.3 (11.2)	17.5 (9.7)	1.07	1.02–1.12	0.004^*^
YGTSS Impairment Score (0–50)	20.0 (15.4)	11.4 (10.9)	1.05	1.02–1.09	0.003^*^

* Candidate predictors in the univariable analysis that met pre-specified statistical criteria for inclusion in the multivariable analysis (nominal *p*-value < 0.1). Responses of “unsure” or “prefer not to answer” were removed prior to analysis, so total sample size varies slightly for each univariable regression.

### 3.5 Correlations between significant predictor variables

Before fitting the multivariable models, each of the candidate predictors that met inclusion criteria from the univariable analyses were examined for pairwise correlations. First, correlations between catamenial mood, anxiety, and OCD changes were calculated using Kendall’s tau. All correlations were statistically significant (all *p* < 0.001). Mood and anxiety changes were highly correlated (*τ* = 0.72). Each other pair had *τ* < 0.50 (Table 3). Next, the relationship between YGTSS Total Tic Score and YGTSS Impairment Score was assessed using Pearson’s correlation. These two variables were also highly correlated (*r* = 0.60, *p* < 0.001).

**Table 3:** Correlations between categorical candidate predictor variables.

Variable	Mood Changes	Anxiety Changes	OCD Changes	Physical Changes
Mood changes	1.00	-	-	-
Anxiety changes	0.72*	1.00	-	-
OCD changes	0.33*	0.34*	1.00	-
Physical changes	0.54*	0.47*	0.37*	1.00

Correlations were calculated using Kendall’s tau after removing unsure/prefer not to answer responses. *p < 0.001.

### 3.6 Multivariable analyses

Due to the strong correlations between catamenial mood and anxiety changes and between YGTSS Total Tic Score and Impairment Score, a single multivariable model would provide unreliable estimates of each predictor’s impact on menstrual cycle-related tic changes. Therefore, four multivariable logistic regression models were fit to represent each combination of the candidate predictor variables (Table 4). Each model included OCD changes, *either* mood (Models 1 and 3) or anxiety changes (Models 2 and 4), and *either* YGTSS Total Tic Score (Models 1 and 2) or Impairment Score (Models 3 and 4) as predictor variables. The four models produced similar results. Catamenial change in OCD symptoms was not significant in any of the models. Change in mood was significantly associated with catamenial tic changes after adjusting for OCD changes and YGTSS Total Tic Score (Model 1; OR = 4.18, *p* = 0.02) or Impairment Score (Model 3; OR = 3.74, *p* = 0.03). Change in anxiety was also significantly associated with catamenial tic changes after adjusting for OCD changes and YGTSS Total Tic Score (Model 2; OR = 3.66, *p* = 0.01) or Impairment Score (Model 4; OR = 3.34, *p* = 0.02). Similarly, YGTSS Total Tic Score was significantly associated with catamenial tic changes after adjusting for OCD changes and mood (Model 1; OR = 1.05, *p* = 0.03) or anxiety changes (Model 2; OR = 1.05, *p* = 0.03). YGTSS Impairment Score was significantly associated with catamenial tic changes after adjusting for OCD changes and mood (Model 3; OR = 1.04, *p* = 0.03) or anxiety changes (Model 4; OR = 1.04, *p* = 0.03).

**Table 4:** Multivariable models of clinical predictors of menstrual cycle-related tic fluctuations.

Model	Variable	OR	95% CI	*p*-value	Nagelkerke Pseudo *R^2^*
Model 1					0.256
	OCD changes	2.55	0.83–7.96	0.10	
	Mood changes	4.18	1.33–16.12	0.02*	
	YGTSS Total Tic Score	1.05	1.01–1.11	0.03*	
Model 2					0.261
	OCD changes	2.54	0.82–7.93	0.11	
	Anxiety changes	3.66	1.35–10.50	0.01*	
	YGTSS Total Tic Score	1.05	1.01–1.11	0.03*	
Model 3					0.257
	OCD changes	2.91	0.97–8.97	0.06	
	Mood changes	3.74	1.19–14.34	0.03*	
	YGTSS Impairment Score	1.04	1.01–1.08	0.03*	
Model 4					0.261
	OCD changes	2.91	0.97–8.88	0.06	
	Anxiety changes	3.34	1.23–9.55	0.02*	
	YGTSS Impairment Score	1.04	1.01–1.08	0.03*	

Due to strong correlations between mood changes and anxiety changes, as well as between YGTSS Total Tic Score and YGTSS Impairment Score, four multivariable models are presented. *p < 0.05. Model 1: *n* = 110, LR *X^2^* (3) = 21.01. Model 2: *n* = 109, LR *X^2^* (3) = 21.31. Model 3: *n* = 110, LR *X^2^* (3) = 21.06. Model 4: *n* = 109, LR *X^2^* (3) = 21.29. All models *p* < 0.001.

Of the four models, Model 2, which included OCD changes, anxiety changes, and YGTSS Total Tic Score, had the best fit, with LR *X^2^* (3) = 21.31 and Nagelkerke pseudo *R^2^* = 0.261 (Table 4). However, the variability in model fit was minimal; the difference between the highest and lowest pseudo *R^2^* across models was 0.005. Therefore, rather than selecting a final model, all four models are presented to suggest that catamenial mood changes, catamenial anxiety changes, YGTSS Total Tic Score, and YGTSS Impairment Score may all play a role in predicting tic changes with menstruation.

## 4 Discussion

The current study suggests that approximately one-fourth of female adults with TS self-report that they experience tic changes at some point in their menstrual cycles, and the vast majority of these changes involve tic symptom worsening. This estimate is consistent with previous studies, which reported catamenial tic fluctuations in 13–34% of female participants (9, 10). However, published research on the menstrual cycle and Tourette syndrome is limited, with only two studies published in the past 35 years. Our study expands upon the previous findings with a substantially larger sample size.

In addition to providing an updated estimate of the prevalence of catamenial tic changes, we determined that two-thirds of participants who reported menstrual cycle-related tic fluctuations experienced these changes exclusively in the premenstrual or luteal phase of the menstrual cycle, while the remainder either experienced tic fluctuations both before and after the first day of their menstrual period (31%) or exclusively after menses onset (3%). These results are consistent with work published by Schwabe and Konkol (9), who observed that the majority of participants with catamenial tic fluctuations reported tic exacerbation in the premenstrual phase (9). The results also align with previous research on OCD and the menstrual cycle, which found that OCD symptoms are increased in the premenstrual period for a subset of individuals (12, 13). These findings suggest that future research should focus on the relationship between neuropsychiatric symptoms and factors that are specific to the premenstrual phase.

Almost two-thirds of female adults with TS reported changes in mood, anxiety, OCD, or other physical symptoms with their menstrual cycles. Mood and anxiety changes as well as tic severity and TS-related impairment in the past 6 months were all identified as significant predictors of self-reported catamenial tic fluctuations. It is difficult to identify which of these associations were most important, in part due to the high correlations between tic severity and impairment and between mood and anxiety changes. Because participants were asked to report these changes retrospectively, some subjects likely found it difficult to distinguish between co-occurring symptoms of mood, anxiety, OCD, and/or tics. This may partially explain the collinearity observed in our results. In addition, we do not know whether our participants’ perceptions of their tic fluctuations accurately represent actual changes in tic symptoms. It is possible that reports of catamenial tic changes are instead explained by menstrual changes in perception, sensitivity, memory, or self-awareness. Similarly, our measures of tic severity and impairment asked participants to report their symptoms over the past 6 months, and were not intended to capture current tic fluctuations in association with the menstrual cycle—instead, these measures are reflective of a general level of severity/impairment at the time of the assessment. We cannot infer causality in any of our analyses—we simply note observed associations. These limitations notwithstanding, our results are of use for two reasons. First, they may be useful for clinicians in helping patients identify factors that may be associated with more severe tic symptoms in adulthood. Second, they do provide an interesting opportunity to examine factors that may be associated with menstrual cycle-related tic symptom changes.

The mechanisms of these changes, particularly in relation to tics, are largely unknown. Previous research on potential contributors to tic worsening—such as sex hormones, neurosteroids, and psychological factors—may give insight into the potential drivers of catamenial tic fluctuations.

Unfortunately, there are few studies in the literature about the effects of sex hormones on tics. A review conducted by Martino et al. (27) suggested that increased exposure to, or activity of, androgenic steroids may exacerbate tic symptoms, though much of the available evidence reported in this review was anecdotal or based on small sample sizes (27). For example, Peterson et al. (28) administered flutamide, a selective androgen receptor antagonist, to 13 adults with TS in a double-blind, placebo-controlled crossover trial. Flutamide reduced the severity of motor (but not phonic) tics, and this improvement faded over time, along with an increase in serum-free testosterone levels (28). Bortolato et al. (29) reported positive effects of finasteride, a 5-alpha reductase (5-αR) inhibitor that blocks the conversion of testosterone to the potent androgen receptor agonist, dihydrotestosterone (DHT), in reducing tic severity in individuals with PMDD (29).

In addition, Martino et al. (27) cited preliminary evidence that the basal activity level of the hypothalamic–pituitary-gonadal (HPG) axis may be reduced in TS patients; however, the hypothalamic–pituitary–adrenal (HPA) axis has also been reported to exhibit enhanced reactivity to external stressors in some individuals with TS (27). This increased responsiveness to stress might result in higher levels of corticotropin-releasing hormone, adrenocorticotropic hormone (ACTH), and cortisol, which could also contribute to tic worsening (30).

Neurosteroids also likely play a role in the neurophysiology that links the menstrual cycle and tic fluctuations. Mosher et al. (31) observed that stress may exacerbate tic-like symptoms by promoting synthesis of the neurosteroid allopregnanolone (AP) from progesterone in the prefrontal cortex of male D1CT-7 transgenic mice, a TS murine model in which dopamine D1 receptor (D1R)-positive neurons have been selectively depleted by activity-dependent expression of a cholera toxin transgene under the regulation of the D1R gene promoter (31). AP functions as a positive GABAergic modulator that activates GABA_A_ receptor activity in response to stressors (29). Another neurosteroid, iso-allopregnanolone (Iso-AP), is a GABA_A_ receptor modulating steroid antagonist (GAMSA) that can both activate and inhibit the GABA_A_ receptor (32). Iso-AP functions in parallel with allopregnanolone to “fine tune” the degree of inhibitory activity in stress-responsive circuits in the brain (33, 34). For example, in the same Mosher et al. study, the authors demonstrated that higher levels of Iso-AP counteracted the increased tic-like symptoms in response to the effects of stress on prefrontal cortex AP levels in D1CT-7 mice, and produced a dose-dependent reduction of tic-like responses to stress (31). Of note, allopregnanolone was recently approved by the FDA to treat PMDD, and Iso-AP formulations are also in clinical trials for PMDD (32, 35).

Research also suggests that, in individuals with PMDD, the GABA_A_ receptor is less sensitive to AP (21). Furthermore, recent studies have shown that reduced levels of AP in the peripheral blood or cerebrospinal fluid are associated with several mood disorders, including major depression, anxiety, and PMDD (36). In some individuals, an increase in AP levels occurring in the midluteal (pre-menstrual) phase of the menstrual cycle leads to elevated mood and anxiety symptoms (37, 38). These findings have led to a growing interest in utilizing both AP and Iso-AP to mediate catamenial neuropsychiatric effects in PMDD. Recent clinical trials have demonstrated that brexanolone, a synthetic formulation of AP (39), significantly reduces depression scores in those with severe postpartum depression in as quickly as 60 hours (40). Further investigation of the therapeutic implications of neurosteroids for tic disorders is needed, particularly for females with TS who experience catamenial tic and/or mood fluctuations.

At a psychological level, the mechanisms underlying patient perception of tic worsening are unclear. Tic worsening in the context of psychosocial stressors has been well documented (41), and anxiety is known to increase the severity of tics and premonitory urges in those with TS (30). It has also been hypothesized that anxiety prompts tic exacerbation only for individuals with a propensity to exhibit externalizing rather than internalizing responses (30). A recent review of 14 studies summarized the evidence for worsening generalized anxiety disorder symptoms in the weeks prior to and following the onset of menses (18), a finding that could implicate worsening anxiety as a mediator of potential hormonal effects on tic worsening during specific phases of the menstrual cycle. Alternatively, it is possible that anxiety could increase sensitivity to tic symptoms, and therefore create the perception of catamenial tic exacerbation, without actually increasing tics. In addition, sleep and circadian rhythms are altered by hormonal changes across the menstrual cycle, and lack of sleep has been correlated with increased tic severity (30, 42). In fact, fatigue can reduce individuals’ tolerance for psychological stressors, thereby compounding the negative effects of stress on tics (30). Finally, sensory overstimulation is also associated with increased tic symptoms, potentially implicating menstrual cycle-related physical changes as a source of perceived tic exacerbation (30).

The current study has several strengths, including the largest sample to date to explore the effects of the menstrual cycle in individuals with TS, and the use of standardized measurements of tic symptom severity and impairment. However, it also has some notable limitations. First, participants were all cisgender females (as established in previous genetic studies with the same participants) and were not asked to report biological sex and gender identity separately. Future studies on catamenial symptom fluctuations should ask participants to report their gender identity and biological sex separately and should include all participants who are menstruating or have menstruated in the past. This would allow for a more gender-diverse sample, which may provide insight into the effects of gender-affirming hormone treatments on catamenial tic fluctuations. Second, participants were not asked about experiences or conditions that can affect the menstrual cycle, such as hormonal contraceptive use, polycystic ovarian syndrome (PCOS), pregnancy, childbirth, or breastfeeding. Future studies should ask about history of pregnancy, childbirth, and breastfeeding, as these factors may influence the menstrual cycle even after pregnancy, and should exclude individuals taking hormonal contraceptives or who have PCOS. Third, White and non-Hispanic participants were greatly overrepresented in the sample. A more diverse sample is necessary to be able to generalize these results to all females with TS and to incorporate culturally dependent experiences of menstruation and symptom fluctuation. Fourth, the questionnaire relied on retrospective recall of symptoms, as we were unable to evaluate symptom changes in real time. Participants, especially those who were post-menopausal, may have remembered their symptoms inaccurately, and therefore our data may be subject to recall bias.

Fifth, we were unable to determine the direction of our findings or assess causality. For example, although we identified a relationship between tic symptom severity and tic fluctuations with the menstrual cycle, we do not know if hormonal changes that cause tic worsening with menses are more likely to be present in those with more severe tics at baseline, whether the hormonal changes cause individuals to experience worse tic severity than others, or if this is a spurious association. Thus, further confirmation of this work using prospective approaches is needed. Sixth, our analyses involving tic medication, OCD diagnosis, ADHD diagnosis, physical changes, TS age of onset, and age at recontact were underpowered to detect a true effect. It is possible that the trends observed in these variables would become significant with a larger sample size. Seventh, there is a potential ascertainment bias related to the 203 subjects that were lost to follow-up. Because we do not know why these individuals chose not to participate in the current study, we cannot perform comprehensive analyses to assess ascertainment bias. Therefore, we need to be cautious when generalizing the findings from this study to all females with TS.

Eighth, participants were only asked if they “noticed that [their] tics change with [their] menstrual cycle,” and were not prompted to report the frequency or intensity of these changes. It is unclear how participants with inconsistent or occasional symptom fluctuations responded to this question. Therefore, it is possible that the proportion of respondents who endorsed menstrual cycle-related tic fluctuations is either an underestimate or an overestimate. Finally, we were unable to assess the magnitude or degree of impairment associated with menstrual cycle-related tic changes, or whether participants experienced these tic changes every month versus only having tic changes during some of their menstrual cycles.

To capture this nuance and to avoid recall bias, future studies would benefit from longitudinal ecological momentary assessments using validated measures that are sensitive to detecting clinically meaningful change (18). Furthermore, serial measurements of estrogen, progesterone, and luteinizing hormone could define the specific phases of the menstrual cycle and the time of ovulation with more precision than day counts (18). Factors such as exposure to life stress could also be measured to assess how stress levels influence tic changes over the menstrual cycle. Finally, future studies of TS and the menstrual cycle should include questions about current and previous hormonal contraceptive use, PCOS, age at menarche, pregnancy, childbirth, breastfeeding, menopause, and the duration and regularity of menstruation.

Despite its limitations, the current study demonstrates that many females with TS experience tic fluctuations with their menstrual cycles. Further research is needed to clarify the direction, magnitude, and frequency of these changes, and to continue investigating the impact of other characteristics and neuropsychiatric symptoms on catamenial tic fluctuations.

## Data Availability

The datasets presented in this article are not readily available because of participant confidentiality. Requests to access the datasets should be directed to the corresponding author: Jeremiah M. Scharf, jscharf@mgb.org.
