# Supplementary material for: Relationships between the menstrual cycle and neuropsychiatric and physical symptoms in females with Tourette syndrome
Source: Front Neurol. 2025 Feb 11;16:1500766. doi: 10.3389/fneur.2025.1500766 (PMC11850270; doi:10.3389/fneur.2025.1500766)
Supplement: Supplementary file 1 [file Supplementary_file_1.docx]

**
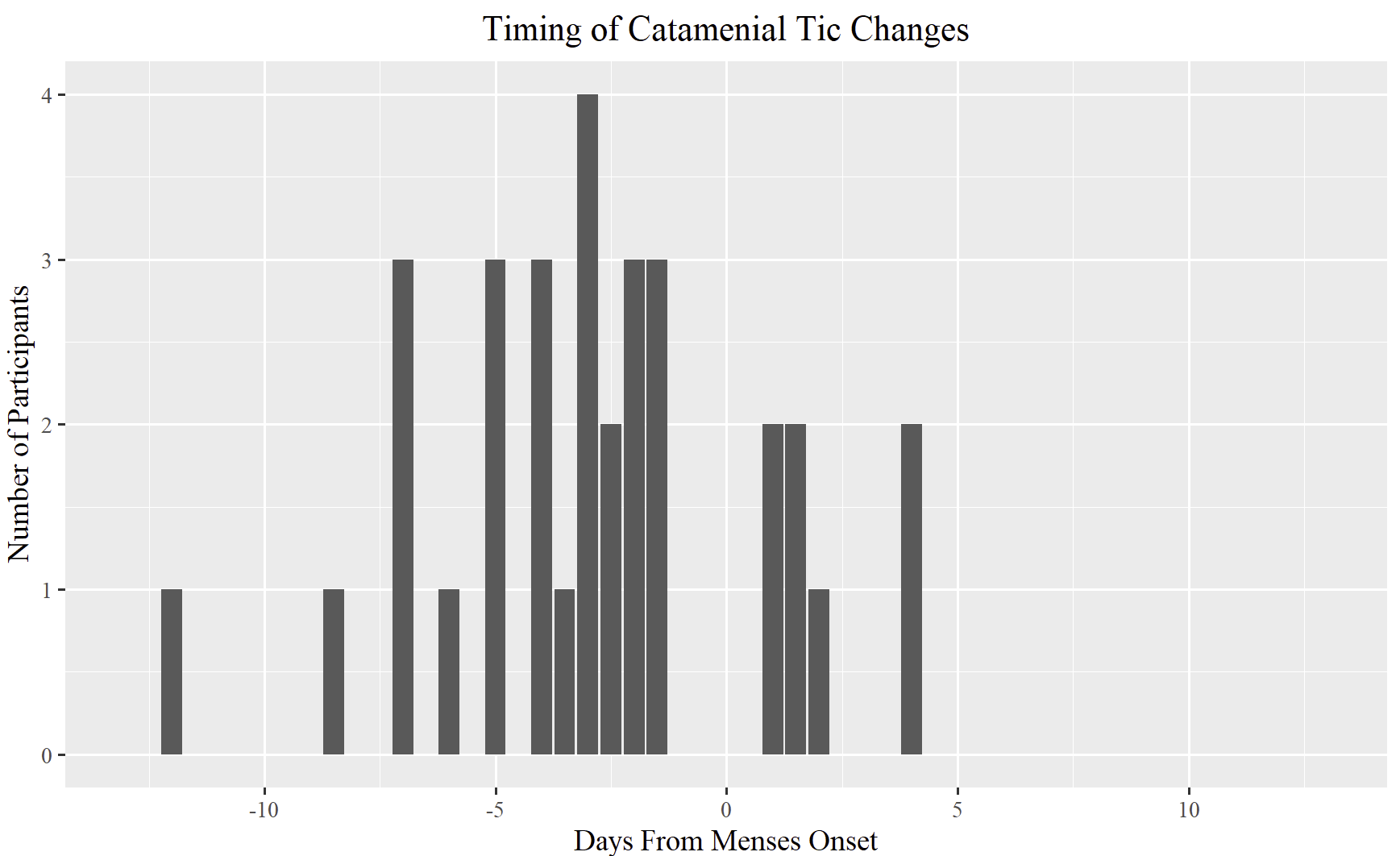
**

**Supplementary Figure 1. Number of days before and after menses onset when tic symptom changes occurred.** For participants who listed a range of days where symptom changes occurred relative to menses onset, the mean of that range was used.
