# Supplementary material for: Relationships between the menstrual cycle and neuropsychiatric and physical symptoms in females with Tourette syndrome
Source: Front Neurol. 2025 Feb 11;16:1500766. doi: 10.3389/fneur.2025.1500766 (PMC11850270; doi:10.3389/fneur.2025.1500766)
Supplement: Supplementary file 5 [file Table_1.docx]

| **Categorical Measures** | | **% Reporting Tic Changes** | **OR** | **95% CI** | ***p*-value** |
| --- | --- | --- | --- | --- | --- |
| Currently taking medication for tics? | Yes (*n* = 29) | 34% | 1.86 | 0.71-4.72 | 0.19 |
|  | No (*n* = 77) | 22% |  |  |  |
| Diagnosed with OCD? | Yes (*n* = 47) | 28% | 1.43 | 0.57-3.64 | 0.45 |
|  | No (*n* = 52) | 21% |  |  |  |
| Diagnosed with ADHD? | Yes (*n* = 30) | 20% | 0.65 | 0.22-1.75 | 0.42 |
|  | No (*n* = 76) | 28% |  |  |  |
| Reported mood, anxiety, OCD, or physical changes? | Yes (*n* = 64) | 38% | 7.80 | 2.47-34.70 | 0.002* |
|  | No (*n* = 42) | 7% |  |  |  |
| Reported mood changes? | Yes (*n* = 61) | 36% | 5.50 | 1.90-20.12 | 0.004* |
|  | No (*n* = 43) | 9% |  |  |  |
| Reported anxiety changes? | Yes (*n* = 42) | 43% | 4.97 | 1.95-13.63 | 0.001* |
|  | No (*n* = 61) | 13% |  |  |  |
| Reported OCD changes? | Yes (*n* = 19) | 53% | 4.79 | 1.68-14.05 | 0.004* |
|  | No (*n* = 85) | 19% |  |  |  |
| Reported physical changes? | Yes (*n* = 31) | 32% | 1.70 | 0.65-4.30 | 0.27 |
|  | No (*n* = 73) | 22% |  |  |  |
| **Continuous Measures** | **Reported Tic Changes Mean (SD)** | **Did Not Report Tic Changes Mean (SD)** | **OR** | **95% CI** | ***p*-value** |
| TS age of onset (years) | 6.7 (2.3) | 7.1 (3.2) | 0.95 | 0.81-1.11 | 0.55 |
| Age at recontact (years) | 32.3 (9.5) | 34.8 (14.0) | 0.98 | 0.95-1.02 | 0.40 |
| YGTSS Total Tic Score (0-50) | 24.4 (11.5) | 17.2 (9.6) | 1.07 | 1.02-1.12 | 0.003* |
| YGTSS Impairment Score (0-50) | 20.0 (15.7) | 10.8 (10.2) | 1.06 | 1.02-1.10 | 0.002* |

**Supplementary Table 1. Sensitivity analysis.** **p*-value < 0.1. Six participants who specifically mentioned taking birth control in an open-ended response question were excluded, resulting in a sample of 106. Responses of “unsure” or “prefer not to answer” were removed prior to analysis, so total sample size varies slightly for each univariable regression.
